# Synergism of *Streptococcus mutans* and *Candida albicans* Reinforces Biofilm Maturation and Acidogenicity in Saliva: An *In Vitro* Study

**DOI:** 10.3389/fcimb.2020.623980

**Published:** 2021-02-19

**Authors:** Hye-Eun Kim, Yuan Liu, Atul Dhall, Marwa Bawazir, Hyun Koo, Geelsu Hwang

**Affiliations:** ^1^ Department of Preventive and Restorative Sciences, School of Dental Medicine, University of Pennsylvania, Philadelphia, PA, United States; ^2^ Department of Orthodontics, School of Dental Medicine, University of Pennsylvania, Philadelphia, PA, United States; ^3^ Center for Innovation & Precision Dentistry, School of Dental Medicine, School of Engineering and Applied Sciences, University of Pennsylvania, Philadelphia, PA, United States

**Keywords:** cross-kingdom biofilm, *Streptococcus mutans*, *Candida albicans*, human saliva, acidogenicity, enamel demineralization

## Abstract

Early childhood caries, a virulent-form of dental caries, is painful, difficult, and costly to treat that has been associated with high levels of *Streptococcus mutans* (Sm) and *Candida albicans* (Ca) in plaque-biofilms on teeth. These microorganisms appear to develop a symbiotic cross-kingdom interaction that amplifies the virulence of plaque-biofilms. Although biofilm studies reveal synergistic bacterial-fungal association, how these organisms modulate cross-kingdom biofilm formation and enhance its virulence in the presence of saliva remain largely unknown. Here, we compared the properties of Sm and Sm-Ca biofilms cultured in saliva by examining the biofilm structural organization and capability to sustain an acidic pH environment conducive to enamel demineralization. Intriguingly, Sm-Ca biofilm is rapidly matured and maintained acidic pH-values (~4.3), while Sm biofilm development was retarded and failed to create an acidic environment when cultured in saliva. In turn, the human enamel slab surface was severely demineralized by Sm-Ca biofilms, while there was minimal damage to the enamel surface by Sm biofilm. Interestingly, Sm-Ca biofilms exhibited an acidic environment regardless of their hyphal formation ability. Our data reveal the critical role of symbiotic interaction between *S. mutans* and *C. albicans* in human saliva in the context of pathogenesis of dental caries, which may explain how the cross-kingdom interaction contributes to enhanced virulence of plaque-biofilm in the oral cavity.

## Introduction

A hyper-virulent form of tooth decay, Early Childhood Caries (ECC), is a biofilm-derived oral disease induced by protracted ingestion of dietary sugars (Hallett and O’Rourke, 2002; Parisotto et al., 2010; Hajishengallis et al., 2017). Interestingly, opportunistic fungal pathogen *Candida albicans* is frequently detected with a cariogenic bacterial pathogen *Streptococcus mutans* in the plaque biofilms formed on the tooth surface of children with ECC (Yang et al., 2012; Xiao et al., 2016; Xiao et al., 2018). Previous *in vitro* and *in vivo* studies demonstrated the mechanism of bacterial-fungal interaction whereby *S. mutans*-derived exoenzymes, glucosyltransferases B (GtfB), avidly bind to *C. albicans* and produce extracellular glucans on *C. albicans* in the presence of sucrose, promoting subsequent *S. mutans* binding to *C. albicans* (Gregoire et al., 2011; Falsetta et al., 2014; Hwang et al., 2015; Hwang et al., 2017). This enhanced interaction accelerates microbial carriage and production of exopolysaccharides (EPS), facilitating enamel dissolution on the tooth surface due to their acidogenic and aciduric characteristics in a milieu rich in dietary carbohydrates (Hwang et al., 2017; Xiao et al., 2017; Pereira et al., 2018).

Human saliva has been recognized to play a significant role in the homeostasis and symbiosis in complex oral environments, contributing to the balance of health and disease (Dawes and Wong, 2019; Pedersen and Belstrøm, 2019). Particularly, saliva exhibited diverse protective functions against dental caries such as i) facilitating clearing irritants, such as microorganisms and dietary carbohydrates; ii) preventing tooth demineralization *via* various inorganics (e.g., calcium and phosphorus), buffering acids from dietary carbohydrates and bacterial fermentation byproducts; iii) providing antimicrobial activity through numerous proteins, peptides (e.g., histidine), and antibodies (secretory immunoglobin A) (Hara and Zero, 2014; van’t Hof et al., 2014; Colombo et al., 2016; Pedersen and Belstrøm, 2019).

As such, saliva can modulate bacterial adhesion and consequent plaque-biofilm formation directly and indirectly. For instance, salivary agglutinin has been found to alleviate *S. mutans* adherence and biofilm formation (Ahn et al., 2008). In contrast, a pellicle formed on the enamel surface *via* adsorption of salivary proteins can facilitate bacterial colonization, resulting in the mature oral biofilm (Cheaib et al., 2015). Specifically, oral streptococci exhibited enhanced binding affinity to the salivary glycoproteins such as mucin and α-amylase (Murray et al., 1992; Cross and Ruhl, 2018). Interestingly, the adhesion of *C. albicans* to the surface of oral streptococci (e.g., *S. gordonii*, *S. oralis*, and *S. sanguinis*) was promoted by selective adsorption of basic salivary proline-rich proteins (O’Sullivan et al., 2000). Although biofilm studies reveal prominent salivary effects on microbial binding and their accumulation to the surface as well as inter-species interaction, there has not been considerable attention on the fitness and behavior of *S. mutans* and *C. albicans* during biofilm development in human saliva in the context of ECC.

Here, we sought to investigate how *S. mutans* and *C. albicans* form mixed-species biofilm and enhance the biofilm virulence in the presence of saliva. To understand the effect of saliva on biofilm behaviors, we comprehensively compared the properties of *S. mutans* single-species (Sm) and *S. mutans-C. albicans* cross-kingdom (Sm-Ca) biofilms cultured in saliva-supplemented media (0–100%). By determining the enamel dissolution caused by these biofilms using ultra-precision surface topography scanning microscopy, we attempted to understand the enhanced virulence of the cross-kingdom biofilms in the context of dental caries. In addition, by testing a hyphal deficient *C. albicans* mutant strain and clinical isolates, we sought to understand how hyphal transformation of *C. albicans* affects the biofilm’s acidogenicity when cultured with *S. mutans* in the presence of saliva. The data reveal an important feature of cross-kingdom biofilm interaction in human saliva, explaining in part why and how these synergistic interactions are deeply involved in ECC.

## Materials and Methods

### Microorganisms and Culture Conditions

*Candida albicans* SC5314, a well-characterized fungal strain, and *Streptococcus mutans* UA159, a proven virulent cariogenic dental pathogen and well-characterized EPS producer, were used for biofilm experiments. *C. albicans* SN152 and its hyphae deficient strain (*efg1ΔΔ*), and two clinical isolates (UR13 and UR18, gift from Jin Xiao, University of Rochester) were also used for evaluating the role of hyphae in biofilm formation (see **Supplementary Table S1** for *C. albicans* strains used in this study). Spider agar plates (1% Difco nutrient broth, 1% mannitol, 0.2% dibasic potassium phosphate, 1.5% agar, pH 7.2) were prepared and used to confirm hyphal deficiency of mutant and clinical isolates of *C. albicans* (Liu et al., 1994). Microbial stocks were stored at −80°C in tryptic soy broth containing 50% glycerol before use. All strains were grown to mid-exponential phase (optical densities at 600 nm of 0.8 and 1.0 for *C. albicans* and *S. mutans*, respectively) in ultrafiltered (10-kDa molecular-mass cutoff; Millipore, Billerica, MA, USA) yeast–tryptone extract broth containing 2.5% tryptone and 1.5% yeast extract (UFYTE; pH 5.5 and 7.0 for *C. albicans* and *S. mutans*, respectively) with 1% (wt/vol) glucose. Cells were harvested by centrifugation (6,000 g, 10 min, 4°C) as described previously (Xiao et al., 2012).

### Saliva Collection

The study protocol was reviewed and approved by the Institutional Review Board of the University of Pennsylvania (protocol #818549). Written informed consent was obtained from all volunteers in this study. Saliva was collected from 4 different healthy individuals (3 females and 1 male) who did not have any medications recently for at least a month. Individual saliva donor chewed unflavored paraffin wax and saliva was collected in a conical tube on ice. This was performed in the morning without having breakfast. Collected saliva was centrifuged (5,500 g, 4°C, 10 min), followed by filter sterilization (0.22 µm polyethersulfone (PES); ultra-low binding protein filter; Millipore, Billerica, MA). Filtered saliva was then kept in a 4°C refrigerator until use.

### *In Vitro* Biofilm Model

Biofilms were formed using our saliva-coated hydroxyapatite (sHA) model as described previously (Koo et al., 2010; Xiao et al., 2012; Falsetta et al., 2014). HA discs (diameter, 1.25 cm; surface area, 2.7 ± 0.2 cm^2^; Clarkson Chromatography Products, Inc., South Williamsport, PA) were coated with filter-sterilized clarified human saliva using 0.22 µm PES filter (Millipore, Billerica, MA) as described previously (Xiao et al., 2012). The HA discs were vertically suspended in 24-well plates using a custom-made wire disc holder, mimicking the free smooth surfaces of the pellicle-coated teeth (Koo et al., 2010; Xiao et al., 2012).

To prepare biofilm culture media in which the proportion of saliva increased in a dose-dependent manner, UFYTE culture medium and filter sterilized saliva were mixed in various ratios; UFYTE:saliva (v/v) 100:0 (SAL_0_), 25:75 (SAL_25_), 50:50 (SAL_50_), and 0:100 (SAL_100_). Each disc was inoculated with i) approximately 2 × 10^6^ CFU of *S. mutans*/ml or ii) ~2 × 10^4^ CFU of *C. albicans*/ml [containing predominantly yeast cell forms (Gregoire et al., 2011)] or iii) both ~2 x 10^6^ CFU of *S. mutans*/ml and ~2 × 10^4^ CFU of *C. albicans*/ml in a prepared culture medium (SAL_0_ to SAL_100_) supplemented with 1% (w/v) sucrose at 37°C under 5% CO_2_; the proportion of the microorganisms in the inoculum is similar to that found in plaque samples from children with ECC (de Carvalho et al., 2006). The culture medium was changed twice daily at 8 am and 6 pm and pH of the supernatant was determined using an Orion pH electrode attached to an Orion DUAL STAR™ pH meter (Thermo Fischer Scientific) until the end of the experimental period (42 h). The biofilms were collected at 18 h and 42 h for imaging and biochemical analysis. At least 3 independent biofilm experiments were performed for each of the conditions.

### Microbiological and Biochemical Biofilm Analysis

Collected biofilms at each time point were subjected to standard microbiological and biochemical analysis. Briefly, the biofilms were removed and homogenized by sonication, and the number of viable cells (total number of CFU per biofilm) was determined (Cocco et al., 2020). In parallel, an aliquot of biofilm suspension was centrifuged (5,500 g, 10 min, 4°C), and the pellet was washed twice with Milli-Q water, dried in an oven (105°C, 24 h), and weighed. Quantification of polysaccharides was performed using an established colorimetric (phenol-sulfuric acid method) assay detailed previously (Klein et al., 2012; Xiao et al., 2012; Falsetta et al., 2014). At least 3 independent biofilm experiments were performed for each of the assays.

To monitor extracellular pH gradients in real-time, 100 μl of the supernatant was collected from biofilm culture media and measured pH at every hour during the middle phase of biofilm culture (18–28 h). In order to harvest the supernatant without affecting biofilm growth nor mechanically disturbing the biofilm, 6 replicates were used. The supernatants were collected from two biofilm samples sequentially at every hour. Total volume of the supernatant collected per biofilm sample does not exceed 300 μl, so that vertically standing sHA discs were not exposed out of the interface of the culture medium.

### Confocal Microscopy Analysis

The biofilms formed in each condition were examined using confocal laser scanning microscopy (CLSM) combined with quantitative computational analysis. Briefly, *S. mutans* cells were stained with 2.5 µM SYTO 9 green-fluorescent nucleic acid stain (485/498 nm; Molecular Probes Inc., Eugene, OR, USA) and *C. albicans* cells were stained with concanavalin A (ConA) lectin conjugated with tetramethylrhodamine at 40 μg/ml (555/580 nm; Molecular Probes, Inc.), while EPS glucans were labeled with 1 µM Alexa Fluor 647-dextran conjugate (647/668 nm; Molecular Probes Inc.) as detailed previously (Xiao et al., 2012; Falsetta et al., 2014). The confocal images of biofilms were obtained using an upright single-photon confocal microscope (LSM800, Zeiss, Jena, Germany) with a 20× (numerical aperture, 1.0) water objective. Each component was illuminated sequentially to minimize cross-talk as follow: SYTO 9 (*S. mutans*) was excited using 488 nm and was collected by a 480/40 nm emission filter; ConA (*C. albicans*) was excited using 560 nm, and was collected by a 560/40 nm emission filter; Alexa Fluor 647 (EPS) was excited using 640 nm and collected by a 670/40 nm emission filter.

To track microbial colonization and subsequent biofilm formation cultured in saliva, biofilm images were taken at 2, 4, 6, 8, and 18 h after seeding microorganisms on the sHA discs in SAL_100_ media. Taken images at each time point were subjected to the quantification of biofilm biomass and visualization. Briefly, image stacks for each channel obtained using a Zeiss LSM800 were converted to 8-bit ome.tiff files and the COMSTAT2 plugin of ImageJ was used to generate values for biovolume (μm^3^/μm^2^). Biovolumes of *S. mutans*, *C. albicans*, and EPS glucans were quantified using COMSTAT2 as detailed elsewhere (Heydorn et al., 2000; Vorregaard, 2008; Kim et al., 2018). Biovolume values for each channel and total biomass up to 8 h were fit to the power-law - V(t) = a×t^b^, where a and b are constants for a particular curve (Paula et al., 2020). At least 3 independent biofilm experiments were performed for each of the analysis.

### Enamel Surface Demineralization

Sterilized human tooth enamel blocks were coated with sterile clarified saliva (sTE, 4mm × 4 mm). ~2 × 10^6^ CFU/ml of *S. mutans* and ~2 × 10^4^ CFU/ml of *C. albicans* were grown on sTE in SAL_100_ containing 1% sucrose (w/v). Biofilms were formed on enamel blocks mounted vertically at 37 °C in 5% CO_2_ for 114 h as described elsewhere (Koo et al., 2010; Liu et al., 2018). SAL_100_ was replaced twice daily. Biofilms were collected at 114 h for standard microbiological and biochemical analysis, and the enamel slabs were collected for topography and surface roughness measurement. The surface topography and roughness of the enamel surface were analyzed by a nondestructive confocal contrasting method using Zeiss LSM 800 with a C Epiplan-Apochromat 50× (numerical aperture, 0.95) nonimmersion objective. The images were processed using ConfoMap (Zeiss) to create 3D topography rendering and measure the surface properties in 3D. To quantify the surface demineralization, 3D surface roughness parameters arithmetical mean height (*S*_a_), maximum peak height (*S*_p_), and maximum pit height (*S*_v_) were measured using ISO 25178 (ISO, 2012). At least 3 independent experiments were performed for the assay.

### Statistical Analyses

All statistical analyses for biochemical, microbiological, biovolume, and topographical data were carried out using GraphPad Prism 8 *via* i) analysis of variance (ANOVA), followed by Dunnett’s test for post-hoc analysis or ii) Student’s t-test where appropriate. The level of significance was set at 5%.

## Results

### Effects of Human Saliva on the Microbiological and Biochemical Properties of Biofilms

**Figure 1:**
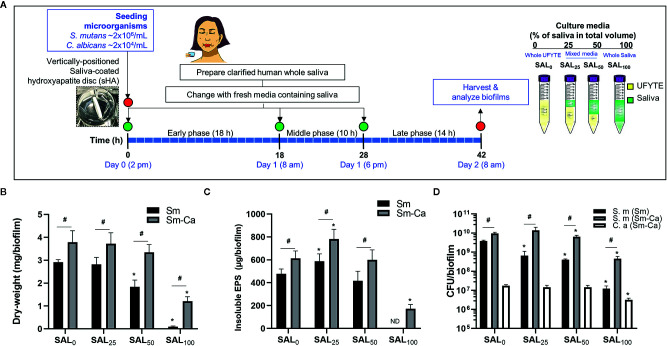
Microbiological and biochemical properties of Sm and Sm-Ca biofilms in saliva contained media (0–100%). **(A)** Biofilm experimental design and composition of culture media. **(B)** Biomass (Dry-weight) of biofilms. **(C)** Amounts of insoluble polysaccharides (EPS) in biofilms. The biomass of Sm biofilm in SAL_100_ was below the detection limit, thus it was not able to measure the content of EPS-glucans in Sm cultured in SAL_100_. **(D)** CFU of *S. mutans* and *C. albicans* in Sm and Sm-Ca biofilms at final phases (42 h). Dry weight, EPS, and CFU experiments were performed (n>3) separately for each donor saliva, and then the average values were calculated. Asterisk indicates that the *p*-values are significantly different from SAL_0_ (whole UFYTE) (ANOVA with Dunnett; *P < 0.05). Hash indicates that the *p*-values are significantly different between two groups (Student’s *t*-test; ^#^ *P* < 0.05). ND indicates not detected.

*S. mutans* (Sm) and *C. albicans* (Ca) exhibit a mutually symbiotic relationship to amplify the virulence of plaque-biofilms (Falsetta et al., 2014; Hwang et al., 2017). However, it remains largely unknown how these organisms modulate cross-kingdom biofilm formation and enhance its virulence in human saliva. To investigate the effect of human saliva on the formation and development of Sm and Sm-Ca biofilms, we prepared bacterial culture media in which the proportion of saliva increased in a dose-dependent manner (SAL_0_ to SAL_100_) and performed biofilm analysis (**Figure 1A**). Clarified human saliva was collected from four donors which presented similar pH buffering capacities (**Supplementary Figure S1**). Dry-weight, EPS, and CFU were assessed individually for each donor saliva (n≥3; **Supplementary Figures S2**-**S5**), and then the average values were calculated (**Figures 1B–D**). We observed that biofilm properties (pH of biofilm supernatant, dry-weight, and CFU) tested by individual saliva were also similar (**Supplementary Figures S2**-**S5**). As shown in **Figure 1**, the dry-weight of Sm biofilm decreased in a dose-dependent manner as saliva content increased and only a negligible amount of biofilm biomass was detected when cultured in 100% clarified human saliva (SAL_100_). However, in the Sm-Ca biofilm, the dry-weight remained unchanged up to 50% of saliva content. Although the dry-weight of Sm-Ca biofilm was reduced significantly in SAL_100_ (vs. 100% UFYTE medium; SAL_0_), a considerable amount of biomass was detected (**Figure 1B**). Total insoluble EPS-glucan (**Figure 1C**) and CFU (**Figure 1D**) also showed similar trends. Note that the biomass of Sm biofilm in SAL_100_ was below the detection limit, thus it was not able to measure the content of EPS glucans in Sm cultured in SAL_100_. Additionally, we investigated the properties of *C. albicans* single-species biofilms in SAL_0_-SAL_100_. The data showed that *C. albicans* alone did not form robust biofilm nor induce acidic pH under all experimental conditions (regardless of saliva concentration in media) (**Supplementary Figure S6**).

### Effect of Saliva on the pH of Biofilm Supernatant

**Figure 2:**
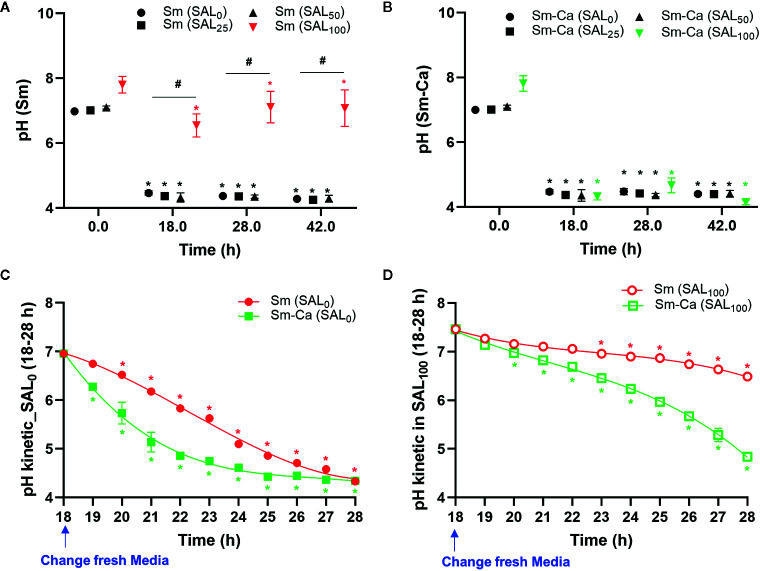
Effect of human saliva on the pH of biofim supernatants in Sm and Sm-Ca biofilms. pH value at early (18 h), middle (28 h), and late (42 h) phases of Sm **(A)** and Sm-Ca **(B)** biofilms in different saliva ratios. Sm and Sm-Ca showed similar pH patterns under most culture conditions (SAL_0_, SAL_25_, and SAL_50_), while Sm biofilm showed a completely different pH pattern when cultured under SAL_100_. Time-lapsed pH measurements of Sm and Sm-Ca biofilms in SAL_0_ **(C)** and SAL_100_ **(D)** during the middle phase (from 18 h to 28 h). Asterisk indicates that the *p*-values are significantly different from the pH of the starting point (ANOVA with Dunnett; *P < 0.05). Hash indicates that the *p*-values are significantly different among groups (Student’s *t*-test; *^#^P* < 0.05).

Dropping of salivary pH under 5.5 is critical for tooth demineralization (Larsen and Pearce, 1997). Thus, we investigated the pHs of the biofilm supernatant throughout the biofilm experimental period (at 18, 28, 42 h). Sm and Sm-Ca biofilms showed very similar levels of pHs in the SAL_0_ media and saliva partially added culture media (SAL_25_ and SAL_50_). However, the pHs of biofilm supernatant in SAL_100_ showed a completely different pattern between Sm and Sm-Ca biofilms (**Figures 2A, B**). Sm-Ca biofilm dropped the pH of biofilm supernatant (~4.3), which was consistent with other cultivation conditions (i.e., SAL_0_-SAL_50_), while Sm biofilm maintained substantially higher pH than the critical point (pH 5.5) that allows enamel demineralization at all time points throughout the biofilm culturing period. Besides, we tracked the dynamic changes of the pH of biofilm supernatant to better understand how these Sm and Sm-Ca biofilms behave in SAL_0_ or SAL_100_ toward biofilm maturation (18–28 h; between 8 am and 6 pm on the Day 2). In SAL_0_, Sm-Ca biofilm more rapidly lowered the pH of biofilm supernatant (vs. Sm), while the pH of both biofilm supernatants reached to similar pH (~4.3) at 28 h (**Figure 2C**). In SAL_100_, Sm-Ca biofilm slowly lowered the pH (vs. SAL_0_) but ultimately reached below pH 5 at 26 h. Although the pH of the Sm biofilm supernatant steadily decreased, it mainly remained in neutral pH (~7) even at 28 h (**Figure 2D**).

### Structural Analysis of Sm and Sm-Ca Biofilms

**Figure 3:**
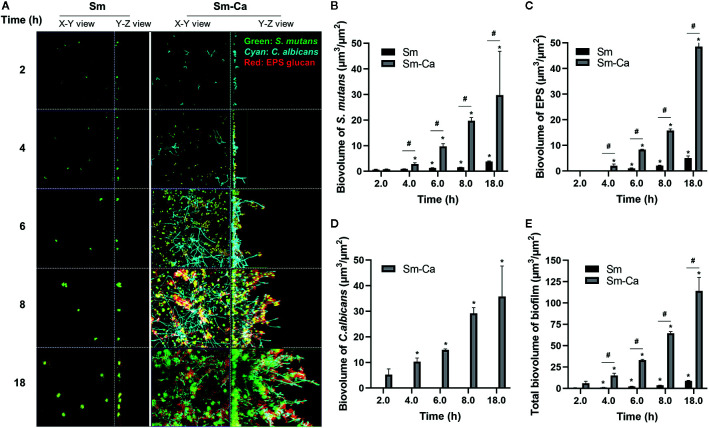
Confocal images of biofilms and quantification of biofilm components. **(A)** Representative top (X–Y) and orthogonal (Y–Z) views of confocal images of Sm and Sm-Ca biofilms at 2, 4, 6, 8, and 18 h cultured under SAL_100_. Bacterial cells are labeled with SYTO 9 (green), fungal cells with concanavalin A-tetramethylrhodamine (Cyan), and EPS α-glucan with Alexa Fluor 647 (red). Quantified biovolume of **(B)** *S. mutans*, **(C)** EPS, **(D)** *C*. *albicans*, and **(E)** total (sum of *S. mutans*, *C*. *albicans*, and EPS) in the biofilm. Asterisk indicates that the *p*-values are significantly different from the biovolume of 2 h (ANOVA with Dunnett; *P < 0.05). Hash indicates that the *p*-values are significantly different between two groups (*t*-test ^#^ *P* < 0.05).

As shown in **Figures 1** and **2**, we observed that only Sm-Ca co-culture resulted in significant biofilm biomass and acidified biofilm supernatant. To further understand differences in biofilm properties between Sm and Sm-Ca biofilms, we investigated the microbial growth and tertiary structure of Sm and Sm-Ca biofilms cultured in SAL_100_ using a confocal microscope (**Figure 3A**). We did not observe a significant difference in *S. mutans* initial binding to the sHA disc between Sm and Sm-Ca biofilms at 2 h (**Figure 3A**). However, we observed distinct growth kinetics of *S. mutans* in Sm and Sm-Ca biofilms at the early stage of biofilm formation (4 h in **Figure 3**, *p*-value: 0.005). This distinct growth kinetics of *S. mutans* between Sm and Sm-Ca biofilms were more pronounced over time (**Figures 3A, B**). Indeed, *S. mutans* biovolume in Sm-Ca biofilm (2.11 µm^3^/µm^2^) was approximately 2.5-fold higher than that of Sm biofilm (0.91 µm^3^/µm^2^) at 4 h (**Figure 3B**). We also observed the explosive production of EPS from Sm-Ca biofilms (**Figure 3C**). After 6 h of inoculation, Sm biofilm still showed only a few numbers of microcolonies with negligible EPS on the sHA disc, while a large number of *S. mutans* microcolonies enmeshed with EPS formed on the sHA disc were observed from Sm-Ca (**Figure 3A**). Growth of *C. albicans* also showed a similar trend with *S. mutans* growth and EPS production (**Figures 3A–D**), resulting in ~12-fold higher total biomass of Sm-Ca at 18 h (vs. Sm).

We have previously shown that biofilm growth dynamics can be fit to power-law curves as a form of V(t) = a×t^b^ (Paula et al., 2020), where V(t) is the biovolume at time t, and a and b are normalization and exponent constants of the curve, respectively. To further elucidate the synergistic interactions in Sm-Ca biofilms, we fitted the biovolume data to the power-law. **Table 1** depicted values for the rate of change of V(t) to highlight the rate of growth, and constants a and b of the curves (up to 8 h). As shown, it is clear that the exponent constant, b, for Sm was ~3.5 times higher in Sm-Ca biofilms than in Sm biofilms. This constant for Sm in single biofilms was under 1, indicating retardation of *S. mutans* growth in SAL_100_. Lastly, the overall constants, a and b, for total biomass in Sm-Ca biofilms were ~7 and ~1.3 times higher, respectively (vs. a and b for total biomass in Sm biofilms).

**Table 1:** Quantified biofilm components of *S. mutans* single-species and *S. mutans-C. albicans* mixed-species biofilms and corresponding power-law parameters.

		*S. mutans*			EPS			*C. albicans*			Total biomass		
**Sm**	Time (h)	ΔV(t)/Δt	a	b	ΔV(t)/Δt	a	b	ΔV(t)/Δt	a	b	ΔV(t)/Δt	a	b
	2	0.31			0.01						0.32		
	4	0.14	0.39	0.65	0.05	6.0E-4	3.94				0.19	0.23	1.27
	6	0.20			0.48						0.68		
	8	0.09			0.53						0.62		
	18	0.22			0.29						0.52		
**Sm-Ca**	Time (h)	ΔV(t)/Δt	a	b	ΔV(t)/Δt	a	b	ΔV(t)/Δt	a	b	ΔV(t)/Δt	a	b
	2	0.32			0.03			2.64			2.99		
	4	0.74	0.11	2.29	0.98	3.8E-3	4.19	2.53	2.21	1.16	4.25	1.75	1.63
	6	2.38			3.18			2.27			7.83		
	8	4.32			3.68			7.13			15.13		
	18	1.00			3.28			0.67			4.94		

Next, we observed the morphology of Sm and Sm-Ca biofilms. Interestingly, the hyphal transition of surface-bound *C. albicans* was initiated from 2 h and these hyphal cells were elongated over time (**Figure 3A**). Particularly, *S. mutans* microcolonies were harmonized with these *C. albicans* in Sm-Ca biofilm, showing numerous small *S. mutans* microcolonies formed along the hyphae (**Figure 3A**, **Supplementary Figure S7**). The data revealed that Sm-Ca synergistic interaction promoted initial biofilm structuring and subsequent development in human saliva.

### Effect of Hyphal Formation Capability of *C. albicans* on Acidogenicity of Cross-kingdom Biofilm

**Figure 4:**
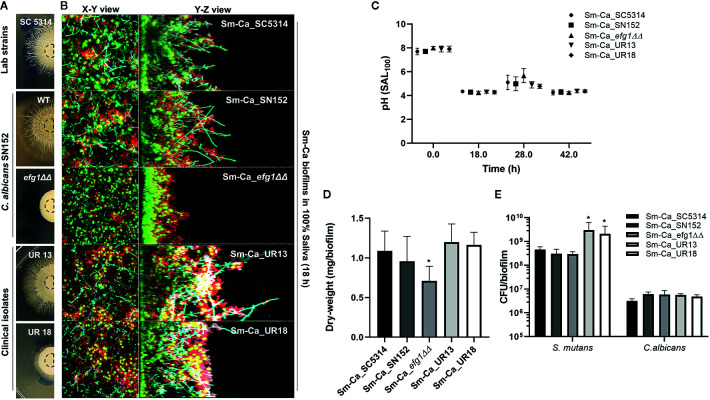
Effect of hyphal transformation ability on the properties of Sm-Ca biofilms in saliva. **(A)** *C*. *albicans* colonies of SC5314, SN152, *efg1ΔΔ*, UR13, and UR18 strains on Spider agar plate. Morphology of fungal colony was photographed after incubation for 4 days at 37°C. **(B)** Representative top (X–Y) and orthogonal (Y–Z) views of confocal images of each mixed-species biofilm under SAL_100_ at 18 h. Bacterial cells are labeled with SYTO 9 (green), fungal cells with concanavalin A-tetramethylrhodamine (Cyan), and EPS α-glucan with Alexa Fluor 647 (red). **(C)** pH value at each time point (18, 28, and 42 h). Data were analyzed by descriptive analysis and one-way analysis of variance (ANOVA) using GraphPad Prism 8. **(D)** Total biomass (dry weight) of each Sm-Ca biofilm and **(E)** CFU of *S. mutans* and *C*. *albicans* at the endpoint (42 h). Asterisk indicates that the *p*-values are significantly different from wild type biofilm (Sm-Ca_SC5314) (ANOVA with Dunnett; *P < 0.05).

Facilitated hyphal elongation of *C. albicans* and the formation of *S. mutans* microcolonies along the hyphae in SAL_100_ may enhance the acidogenicity of Sm-Ca biofilm (**Figure 2**). To investigate the role of the hyphal formation capability of *C. albicans* on Sm-Ca biofilm development in SAL_100_, we compared the biofilm properties between *C. albicans* wild type (Ca_WT; SC5314 or SN152) and its hyphal-deficient mutant strain EFG1 (Ca*_efg1ΔΔ*) (**Supplementary Table S1**). First, we confirmed the hyphal formation ability Ca*_efg1ΔΔ* using spider agar plates. As expected, Ca_WT formed dense hyphae, while the Ca*_efg1ΔΔ* strain did not form hyphae at all on spider agar plates (**Figure 4A**). We also examined the morphologies of biofilms formed by these two *C. albicans* strains and *S. mutans*. Representative confocal images of 18 h biofilm clearly showed that Ca*_efg1ΔΔ* strain has no ability to transform yeast to hyphae even when cultured with *S. mutans* in SAL_100_. In contrast, Sm-Ca_WT showed abundant long-hyphae across the surface (**Figure 4B**). Also, Sm-Ca_*efg1ΔΔ* formed a flat and thinner biofilm in SAL_100_, resulting in significantly less biomass (vs. Sm-Ca_WT; **Figure 4D**). Nevertheless, there were no statistical differences in CFU and more importantly pH profiles, regardless of strains (**Figure 4C**).

To gain further insight into the effect of hyphal transition on the biofilm formation and acidogenicity, we also tested two clinical isolates of *C. albicans* collected from ECC patients. We selected these two isolates as it showed clear differences in hyphal development efficiencies (UR13: long hyphae; UR18: short hyphae on spider agar plates, **Figure 4A**). Both Sm-Ca_UR13 and Sm-Ca_UR18 exhibited acidic microenvironment (pH<4.5), similar to other cross-kingdom biofilm combinations (**Figure 4C**). Dry-weight of biofilms formed with clinical isolates also showed a similar level to the one from Sm-Ca_WT (**Figure 4D**). Interestingly, *S. mutans* population in Sm-Ca_UR13 (or Sm-Ca_UR18) was approximately a log higher than other combinations, while there were no significant differences in *C. albicans* populations (**Figure 4E**). The data indicated that the lack of yeast-to-hyphae transition of *C. albicans* in the cross-kingdom biofilm did not compromise the acidogenicity of biofilms.

### Demineralization of Human Enamel Surface by Biofilms Cultured in Human Saliva

**Figure 5:**
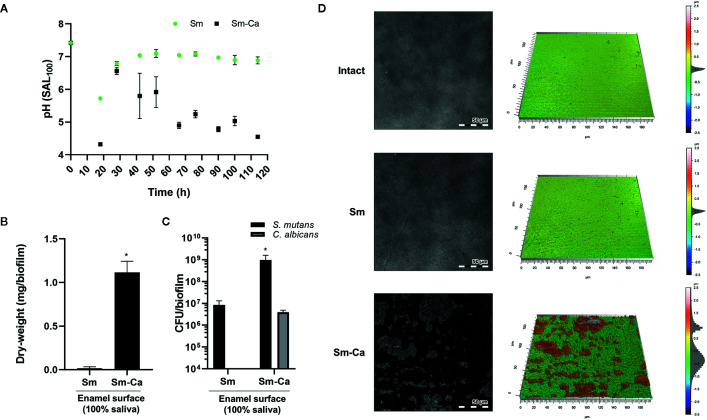
Demineralization of human enamel surface by Sm and Sm-Ca biofilm in SAL_100_. **(A)** pH changes in Sm and Sm-Ca biofilm throughout the biofilm experiment. **(B)** Biomass (Dry-weight) of biofilms and **(C)** CFU of *S. mutans* and *C*. *albicans* in Sm and Sm-Ca biofilms at final phases (114 h). **(D)** Representative confocal surface-topography and roughness of enamel surfaces (Scale bar: 50 µm). Asterisk indicates that the *p*-values are significantly different from Sm biofilm (Student’s *t*-test; *P < 0.05).

Finally, we compared the level of enamel demineralization by culturing each biofilm on the human enamel slab in SAL_100_ supplemented with 1% sucrose. As shown in **Figure 5A**, we observed a distinct difference in the pH of biofilm supernatant between Sm and Sm-Ca biofilms (**Figure 5A**). Sm biofilm hardly lowered the pH of biofilm supernatant as we observed from the previous experiment using HA disc. In contrast, Sm-Ca biofilm gradually lowered the pH of biofilm supernatant over time; eventually, it reached acidic pH below 5.5 from 66 h till the end of the experiment (**Figure 5D**). By culturing biofilms for five days on human enamel slabs, we were able to have similar biofilm biomass and CFU to the one we observed from the HA disc model (**Figures 4D, E** and **5B, C**). Such significant differences in pH and biofilm density between Sm and Sm-Ca biofilms could inflict differential damage to the mineralized tooth tissue underneath the distinctive biofilms. Thus, we inspected the enamel destruction by biofilms visually and quantitatively using confocal surface topographical analysis. As shown in **Figure 5D**, the intact surface before forming biofilms showed a smooth and flat surface with subnanometer-level surface roughness (**Figures 5D, E**). We also observed mostly intact but mildly damaged surfaces where Sm biofilm was formed (**Figures 5D, E**). In marked contrast, the enamel surfaces accommodated Sm-Ca biofilm showed significantly eroded surfaces with microcavities (**Figure 5D**). This observation was supported by quantitative analysis of arithmetical mean height (*S*_a_), maximum peak height (*S*_p_), and maximum pit height (*S*_v_) following ISO 25178 (ISO, 2012). Overall, enamel surfaces degraded by Sm-Ca biofilms exhibited ~6-fold higher *S*_a_, ~2.8-fold higher *S*_p_, and ~1.7-fold higher *S*_v_, than those degraded by Sm biofilms (**Table 2**). Collectively, our findings clearly show that the symbiotic and synergistic interactions between *S. mutans* and *C. albicans* are critical to forming mature biofilms in human saliva that can result in localized enamel demineralization and tooth decay.

**Table 2:** Surface roughness parameters of the demineralization of human enamel by biofilms.

Surface Roughness Parameter (µm)	Intact	Sm	Sm-Ca
*S_a_* (arithmetical mean height)	0.0388 ± 0.0098	0.1347 ± 0.0521	0.7880 ± 0.5087
*S_p_* (maximum peak height)	1.0810 ± 0.5863	3.6960 ± 4.5459	10.2978 ± 2.8721
*S_v_* (maximum pit height)	1.6482 ± 1.1531	3.6224 ± 3.1856	6.0872 ± 3.2119

## Discussion

Plenty of studies have shown that plaque biofilms cause various infectious oral diseases in the human mouth (Marsh, 2010; Murakami et al., 2018). Specific to dental caries, high levels of *S. mutans* and *C. albicans* are found from plaque-biofilms in ECC patients (de Carvalho et al., 2006; Xiao et al., 2018). These microbes develop symbiotic cross-kingdom interactions that amplify the toxicity of plaque-biofilms (Falsetta et al., 2014; Hwang et al., 2017). Although saliva plays critical roles in the oral cavity such as inhibiting microbial activity or promoting microbial adhesion (Ahn et al., 2008; Cheaib et al., 2015), studies on the effect of saliva on the behaviors and fitness of cross-kingdom biofilm have received limited attention.

Here, we compared the biofilm properties cultured in saliva contained media (0 to 100%) and investigated how human saliva modulates the acidogenicity and virulence of cariogenic cross-kingdom biofilms to mimic physiological conditions in the human mouth. While both Sm and Sm-Ca biofilms prospered and created acidic microenvironments either in an optimized culture medium (SAL_0_) or saliva-supplemented media (SAL_25_ or SAL_50_), Sm biofilm was more susceptible to saliva contents; dry-weight started to substantially reduce when saliva proportion was higher than 50% and it was barely detected in the 100% clarified saliva without exhibiting acidogenicity (**Figure 2A**). Although, we also observed a significant reduction of dry-weight of Sm-Ca biofilm in SAL_100_, Sm-Ca biofilm matured sufficiently to induce an acidic environment (**Figure 2B**). The data revealed distinct biofilm fitness in response to saliva concentration, thus it is imperative to more closely mimic clinically relevant conditions when performing *in vitro* study. By tracking initial settlement and colonization of microbes on the sHA discs over time, we found that the colonized *S. mutans* in Sm-Ca biofilm on the sHA disc were more rapidly grown in SAL_100_ (vs. Sm; **Figure 3A**). In turn, acidogenicity of the Sm-Ca biofilm was reinforced, thereby incurring severe enamel dissolution (**Figure 5D**).

Saliva contains a wide variety of antimicrobial peptides (AMPs) that act as host defense molecules (such as defensins and histatins) to combat microbial infestations and challenges (Helmerhorst et al., 1999; Dale and Fredericks, 2005; Jurczak et al., 2015). Therefore, we examined whether the 100% clarified saliva could inhibit the viability and growth of *S. mutans*, thereby blocking them from forming mature biofilm in SAL_100_. Interestingly, human saliva was no harm to *S. mutans* in the planktonic phase. Rather, it showed a similar *S. mutans* population either in single-species or mixed-species cultivation (**Supplementary Figure S8**). A similar observation has been reported that *S. mutans* was resistant to AMPs in saliva (Phattarataratip et al., 2011). Although saliva did not affect the viability of *S. mutans*, *S. mutans* alone failed to form mature biofilm in SAL_100_, while significant biomass was detected from Sm-Ca biofilm under the same conditions (**Figure 1B**). These results suggest that saliva may affect further biofilm development on the surface. Dramatic increases in V(t)/Δt (>>1) of all biofilm components in Sm-Ca indicate their explosive growth at the initial developmental phase. In contrast, V(t)/Δt of all those components in Sm showed significantly less than 1, indicating that their growth was saturating over time (**Table 1**). The data revealed that the co-existence of *S. mutans* and *C. albicans* significantly enhanced the growth kinetics of Sm-Ca biofilm in SAL_100_ (vs. Sm).

*C. albicans* undergoes a reversible morphological transition from single yeast cells to pseudohyphae and hyphae filaments (Carlisle and Kadosh, 2013). Hyphae of *C. albicans* are essential elements for the structural integrity to be fully developed biofilms with multilayered architectures (Baillie and Douglas, 1999; Kadosh, 2013). By investigating the properties of diverse cross-kingdom biofilms using hyphal deficient *C. albicans* strains, we confirmed that all the cross-kingdom biofilms tested in this study could sufficiently lower the acidity of biofilm (< pH 4.5) that can cause enamel demineralization, regardless of the hyphal formation capability (**Figure 4**). Although we encountered a significant reduction in biofilm biomass when cultured with complete hyphal defective strain Ca_*efg1ΔΔ* in SAL_100_, interestingly, there were no significant differences in *S. mutans* population nor pHs of biofilm supernatant (**Figures 4C, D**). These results suggest that the hyphal transition of *C. albicans* induced by human saliva may facilitate the structural maturation of Sm-Ca biofilms (vs. Sm-Ca_*efg1ΔΔ*); the formation of small microcolonies along the hyphae during biofilm development might contribute to enhanced biofilm thickness of Sm-Ca biofilms (**Supplementary Figure S6**). Nevertheless, the alliance between *S. mutans* and yeast form of *C. albicans* in Sm-Ca_*efg1ΔΔ* was sufficient to surge their acidification. Since *S. mutans* binds to mannan in *C. albicans* cell wall *via* surface-formed glucans (Hwang et al., 2017), their symbiotic interaction could be less affected by the hyphal formation of *C. albicans*.

In addition, a previous study on metabolites/chromatographic analyses of bacterial-fungal derived conditioned medium revealed that it contained elevated amounts of organic acids; formate concentration was significantly increased in biofilms and *S. mutans gtf*BC expression was significantly enhanced which is essential for microcolony development (Kim et al., 2017). In our study, the accumulation of Sm-Ca biofilms on human enamel slab resulted in its erosion and severe alteration of surface topographies (**Figure 5D**). Thus, it is conceivable that Sm-Ca biofilms in saliva upregulated acid production (vs. Sm) thanks to synergistic interactions, thereby accelerating tooth decay. Therefore, it is necessary to investigate the metabolite composition in each biofilm cultured in saliva and analyze gene expressions associated with their metabolic activity during biofilm culturing to further understand the mechanism of action. Additionally, further studies regarding the role of salivary components on cross-kingdom interaction are required to fully understand these phenomena.

In summary, by performing *in vitro* experiments under more clinically relevant conditions (utilizing clarified human saliva), we provide feasible data to support the clinical findings in regards to the synergistic association between *S. mutans* and *C. albicans* in the pathogenesis of ECC. A key finding of this study is that the presence of *C. albicans* is critical to enhancing the maturity of biofilm and sustaining an acidic environment with *S. mutans* conducive to enamel demineralization in the 100% clarified saliva. While the hyphal transition of *C. albicans* assists further development of biofilm thickness, it is not essential for the acidogenicity of cross-kingdom biofilms. Our data explain, at least partly, why these organisms and rampant carious lesions are frequently observed from the plaque and teeth of children afflicted with ECC. Further understanding of the molecular biology mechanisms for *S. mutans-C. albicans* cross-talk in human saliva may lead to novel therapeutics to prevent and disrupt costly dental caries, such as ECC.

## Data Availability Statement

The original contributions presented in the study are included in the article/supplementary material. Further inquiries can be directed to the corresponding author.

## Ethics Statement

The studies involving human participants were reviewed and approved by the Institutional Review Board of the University of Pennsylvania (protocol #818549). The patients/participants provided their written informed consent to participate in this study.

## Author Contributions

GH planned and constructed the study. H-EK, YL, AD, MB, HK, and GH contributed to data acquisition and interpretation. H-EK and AD performed all statistical analyses. H-EK, YL, AD, and GH drafted the manuscript and critically revised the manuscript. All authors contributed to the article and approved the submitted version.

## Funding

This work was supported in part by the National Institutes for Dental and Craniofacial Research (NIDCR) grants DE027970 (GH) and DE025220 (HK).

## Conflict of Interest

The authors declare that the research was conducted in the absence of any commercial or financial relationships that could be construed as a potential conflict of interest.
