# Supplementary material for: Synergism of Streptococcus mutans and Candida albicans Reinforces Biofilm Maturation and Acidogenicity in Saliva: An In Vitro Study
Source: Front Cell Infect Microbiol. 2021 Feb 19;10:623980. doi: 10.3389/fcimb.2020.623980 (PMC7933670; doi:10.3389/fcimb.2020.623980)
Supplement: Supplementary file 1 [file DataSheet_1.docx]

**Supplemental information for ‘Synergism of *Streptococcus mutans* and *Candida albicans* reinforces biofilm maturation and acidogenicity in saliva: an *in vitro* study’**

Hye-Eun Kim^1^, Yuan Liu^2^, Atul Dhall^1^, Marwa M. Bawazir^1^, Hyun Koo^2,3^, Geelsu Hwang^1,3*^

^1^Department of Preventive and Restorative Sciences, School of Dental Medicine, University of Pennsylvania, Philadelphia, PA 19104, USA

^2^Department of Orthodontics, School of Dental Medicine, University of Pennsylvania, Philadelphia, PA 19104, USA

^3^Center for Innovation & Precision Dentistry, School of Dental Medicine, School of Engineering and Applied Sciences, University of Pennsylvania, Philadelphia, PA 19104, USA

Corresponding Author:

* Geelsu Hwang; email: [geelsuh@upenn.edu](mailto:geelsuh@upenn.edu)

Supplementary Figure S1. pH buffering capacities of saliva from each donor.


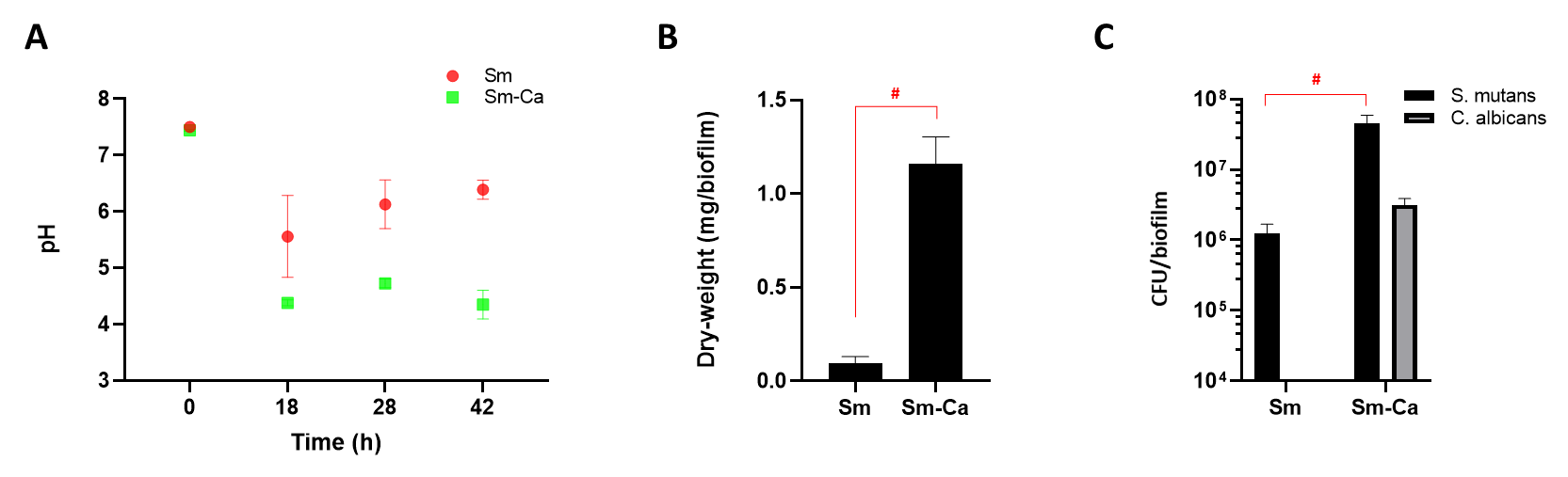


Supplementary Figure S2. Microbiological and biochemical properties of Sm and Sm-Ca biofilms cultured in human saliva (Donor 1). (A) pH value at early (18 h), middle (28 h), and late (42 h) phases in Sm and Sm-Ca biofilms. (B) Biomass (Dry-weight) of biofilms and (C) CFU of *S. mutans* and *C. albicans* in Sm and Sm-Ca biofilms at final phases. Hash indicates that the p-values are significantly different between two groups (^#^*P* < 0.05).


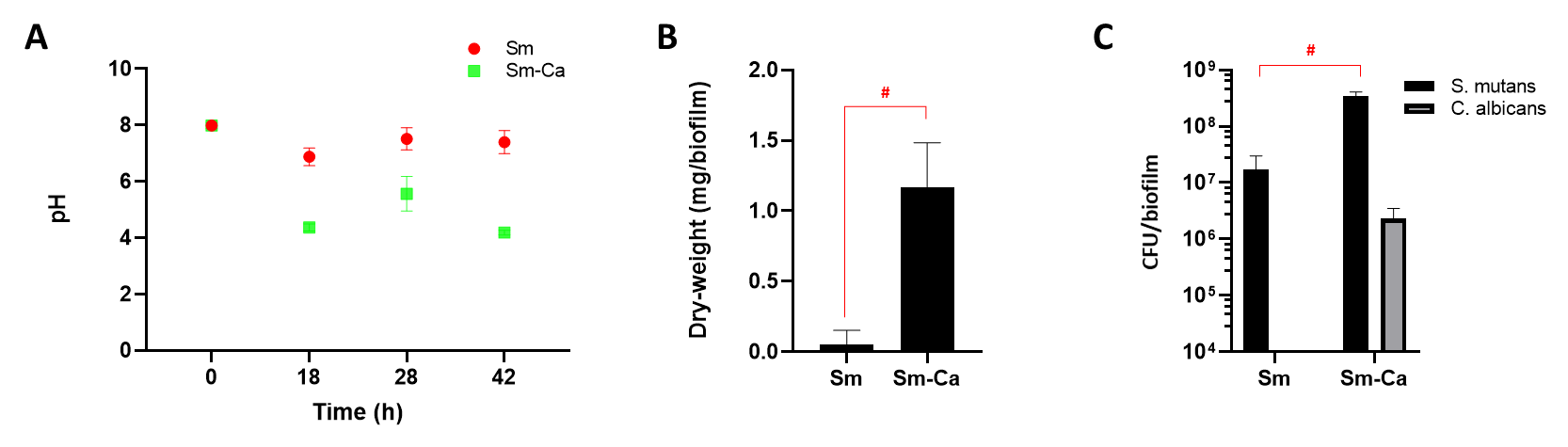


Supplementary Figure S3. Microbiological and biochemical properties of Sm and Sm-Ca biofilms cultured in human saliva (Donor 2). (A) pH value at early (18 h), middle (28 h), and late (42 h) phases in Sm and Sm-Ca biofilms. (B) Biomass (Dry-weight) of biofilms and (C) CFU of *S. mutans* and *C. albicans* in Sm and Sm-Ca biofilms at final phases. Hash indicates that the p-values are significantly different between two groups (^#^*P* < 0.05).


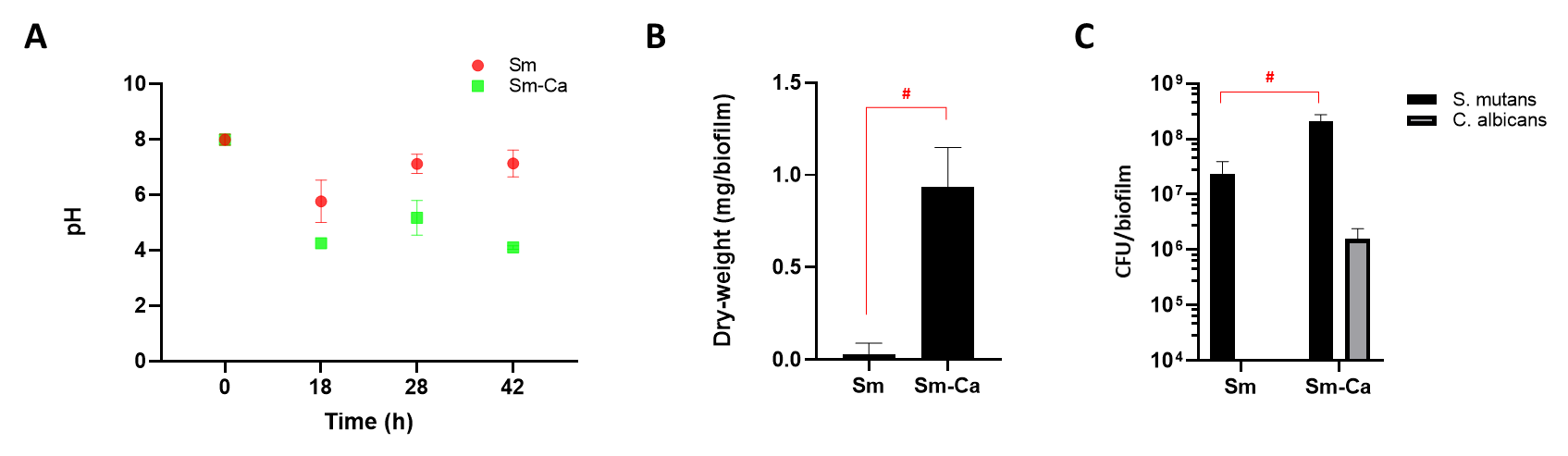


Supplementary Figure S4. Microbiological and biochemical properties of Sm and Sm-Ca biofilms cultured in human saliva (Donor 3). (A) pH value at early (18 h), middle (28 h), and late (42 h) phases in Sm and Sm-Ca biofilms. (B) Biomass (Dry-weight) of biofilms and (C) CFU of *S. mutans* and *C. albicans* in Sm and Sm-Ca biofilms at final phases. Hash indicates that the p-values are significantly different between two groups (^#^*P* < 0.05).


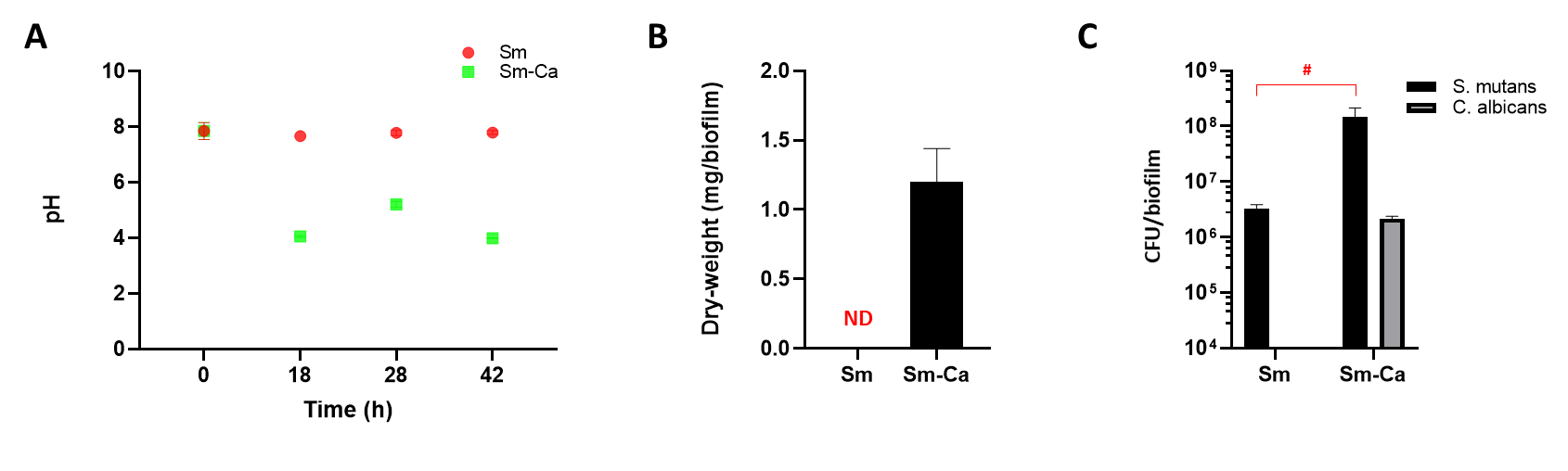


Supplementary Figure S5. Microbiological and biochemical properties of Sm and Sm-Ca biofilms cultured in human saliva (Donor 4). (A) pH value at early (18 h), middle (28 h), and late (42 h) phases in Sm and Sm-Ca biofilms. (B) Biomass (Dry-weight) of biofilms and (C) CFU of *S. mutans* and *C. albicans* in Sm and Sm-Ca biofilms at final phases. ND: not detected. Hash indicates that the p-values are significantly different between two groups (^#^*P* < 0.05).


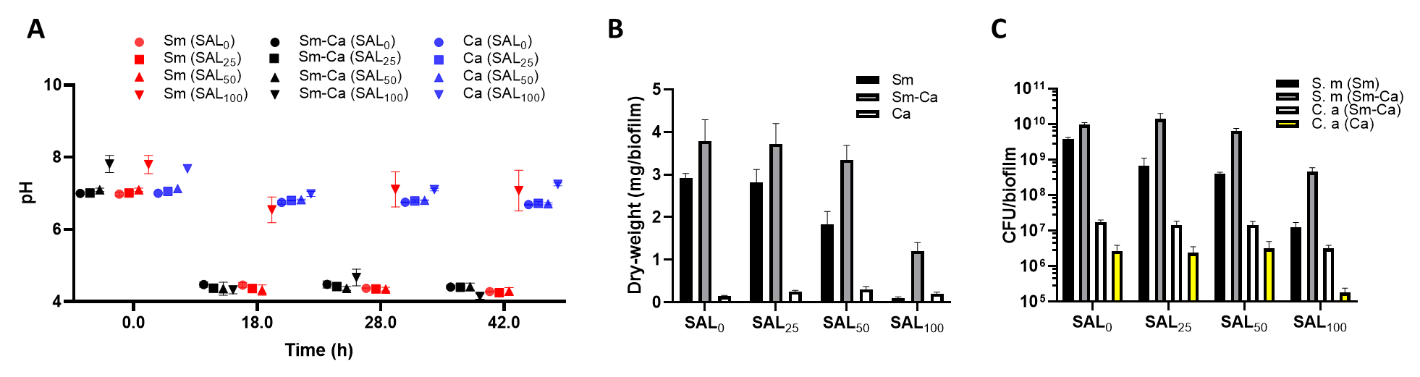


Supplementary Figure S6. Microbiological and biochemical properties of Sm, Ca, and Sm-Ca biofilms cultured in saliva-supplemented media (SAL_0_-SAL_100_). (A) pH value at early (18 h), middle (28 h), and late (42 h) phases. (B) Biomass (Dry-weight) of biofilms and (C) CFU of *S. mutans* and *C. albicans* in each biofilm at final phases.


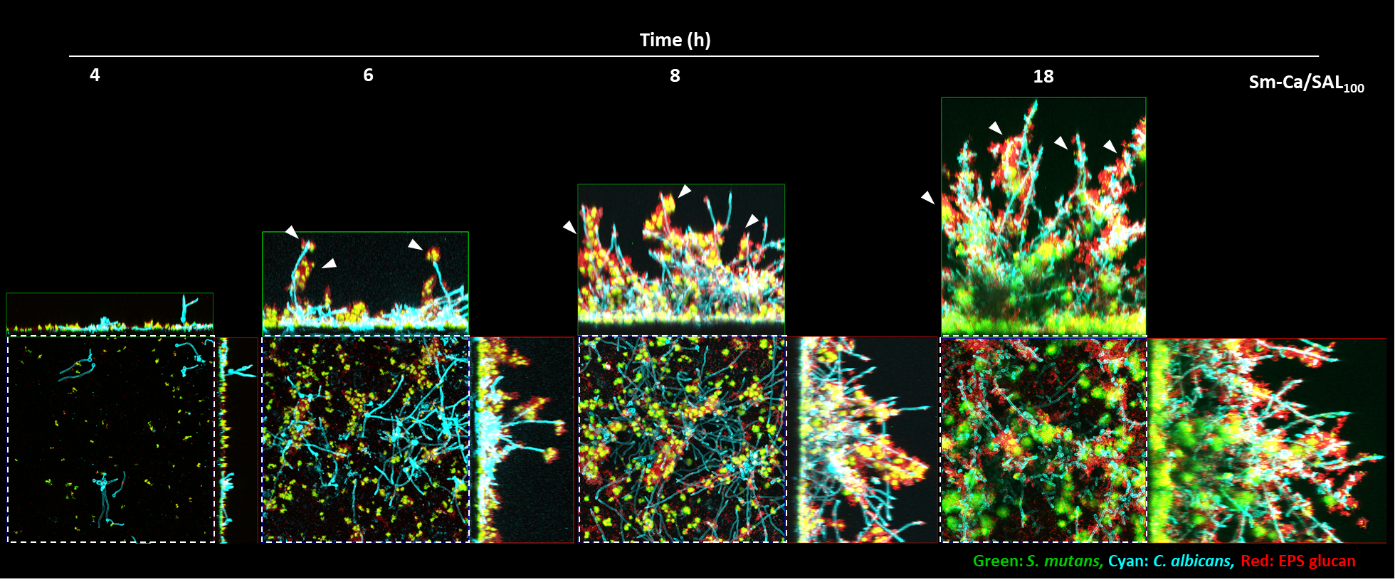


Supplementary Figure S7. Representative top and orthogonal views of confocal images of Sm and Sm-Ca biofilms over time under SAL_100_ (4, 6, 8, and 18 h). White arrows indicate that numerous small *S. mutans* microcolonies were formed along the hyphae.


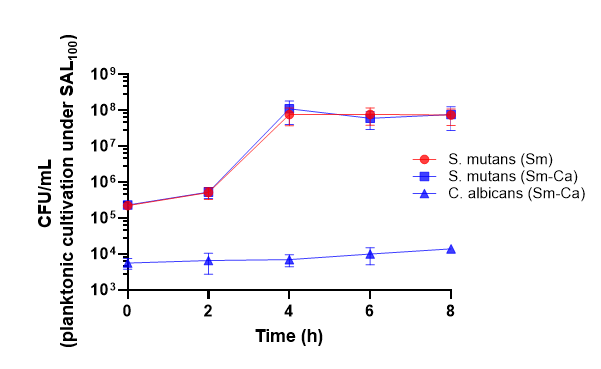


Supplementary Figure S8. Changes in CFU of *S. mutans* and *C. albicans* in Sm and Sm-Ca biofilms in planktonic cultivation under human whole saliva (SAL_100_).

| **Strain/Collection** | **Genotype** | **Hyphae** | **References** |
| --- | --- | --- | --- |
| SC5314 | Wild type | Normal | [1] |
| SN152 | As CAI4 butarg4Δ/*arg4Δ leu2Δ/leu2Δ his1Δ/his1Δ IRO1/iro1Δ*::*imm434* | Normal | [2] |
| *efg1∆∆* | ura3::imm434/ura3::imm434 efg1::hisG/efg1::hisG::URA3::hisG | Deficient | [3] |
| UR13 | Clinical isolates from plaque biofilm of ECC patients | Normal | In this study^a^ |
| UR18 | Clinical isolates from plaque biofilm of ECC patients | Reduced | In this study^a^ |

Supplementary Table S1. *C. albicans* strains used in this study.

^a^ Two clinical isolates, UR13 and UR18, are gift from Jin Xiao, University of Rochester.
